# Discovery and fine-mapping of loci associated with MUFAs through trans-ethnic meta-analysis in Chinese and European populations[S]

**DOI:** 10.1194/jlr.P071860

**Published:** 2017-04-28

**Authors:** Yao Hu, Toshiko Tanaka, Jingwen Zhu, Weihua Guan, Jason H. Y. Wu, Bruce M. Psaty, Barbara McKnight, Irena B. King, Qi Sun, Melissa Richard, Ani Manichaikul, Alexis C. Frazier-Wood, Edmond K. Kabagambe, Paul N. Hopkins, Jose M. Ordovas, Luigi Ferrucci, Stefania Bandinelli, Donna K. Arnett, Yii-Der I. Chen, Shuang Liang, David S. Siscovick, Michael Y. Tsai, Stephen S. Rich, Myriam Fornage, Frank B. Hu, Eric B. Rimm, Majken K. Jensen, Rozenn N. Lemaitre, Dariush Mozaffarian, Lyn M. Steffen, Andrew P. Morris, Huaixing Li, Xu Lin

**Affiliations:** The Key Laboratory of Nutrition and Metabolism,* Institute for Nutritional Sciences, Shanghai Institutes for Biological Sciences, Chinese Academy of Sciences, University of the Chinese Academy of Sciences, Shanghai, People’s Republic of China; Translational Gerontology Branch,† National Institute on Aging, Baltimore, MD; Division of Biostatistics§ University of Minnesota, Minneapolis, MN; Division of Epidemiology and Community Health,‖‖‖‖ School of Public Health, University of Minnesota, Minneapolis, MN; Department of Laboratory Medicine and Pathology,**** University of Minnesota, Minneapolis, MN; George Institute for Global Health,‡ Sydney Medical School, University of Sydney, Sydney, New South Wales, Australia; Cardiovascular Health Research Unit, Department of Medicine,‖ University of Washington, Seattle, WA; Department of Biostatistics,$ University of Washington, Seattle, WA; Department of Epidemiology, School of Public Health,†††† University of Washington, Seattle, WA; Group Health Research Institute,# Group Health Cooperative, Seattle, WA; Department of Internal Medicine,** University of New Mexico, Albuquerque, NM; Channing Division of Network Medicine,†† Department of Medicine, Brigham and Women’s Hospital and Harvard Medical School, Boston, MA; Department of Nutrition,§§ Harvard T. H. Chan School of Public Health, Harvard University, Cambridge, MA; Institute of Molecular Medicine,‡‡ University of Texas Health Science Center at Houston, Houston, TX; Center for Public Health Genomics,‖‖ University of Virginia, Charlottesville, VA; Biostatistics Section, Department of Public Health Sciences,## University of Virginia, Charlottesville, VA; USDA Children’s Nutrition Research Center,$$ Department of Pediatrics, Baylor College of Medicine, Houston, TX; Division of Epidemiology,*** Department of Medicine, Vanderbilt University Medical Center, Nashville, TN; Department of Internal Medicine, ††† University of Utah, Salt Lake City, UT; Nutrition and Genomics Laboratory, Jean Mayer-USDA Human Nutrition Research Center on Aging,§§§ Tufts University, Boston, MA; Friedman School of Nutrition Science and Policy,‡‡‡‡ Tufts University, Boston, MA; Department of Epidemiology and Population Genetics,‡‡‡ National Center for Cardiovascular Investigation, Madrid, Spain; Geriatric Unit,‖‖‖ Azienda Sanitaria Firenze, Florence, Italy; Department of Epidemiology,### University of Alabama at Birmingham, Birmingham, AL; Institute for Translational Genomics and Population Sciences,$$$ Los Angeles BioMedical Research Institute at Harbor-UCLA Medical Center, Torrance, CA; New York Academy of Medicine,§§§§ New York, NY; Genetic and Genomic Epidemiology Unit,#### Wellcome Trust Centre for Human Genetics, Oxford, United Kingdom

**Keywords:** genetics, fatty acid/desaturases, fatty acid/metabolism, fatty acid/biosynthesis, monounsaturated fatty acid

## Abstract

MUFAs are unsaturated FAs with one double bond and are derived from endogenous synthesis and dietary intake. Accumulating evidence has suggested that plasma and erythrocyte MUFA levels are associated with cardiometabolic disorders, including CVD, T2D, and metabolic syndrome (MS). Previous genome-wide association studies (GWASs) have identified seven loci for plasma and erythrocyte palmitoleic and oleic acid levels in populations of European origin. To identify additional MUFA-associated loci and the potential functional variant at each locus, we performed ethnic-specific GWAS meta-analyses and trans-ethnic meta-analyses in more than 15,000 participants of Chinese and European ancestry. We identified novel genome-wide significant associations for vaccenic acid at *FADS1/2* and *PKD2L1* [log_10_(Bayes factor) ≥ 8.07] and for gondoic acid at *FADS1/2* and *GCKR* [log_10_(Bayes factor) ≥ 6.22], and also observed improved fine-mapping resolutions at *FADS1/2* and *GCKR* loci. The greatest improvement was observed at *GCKR*, where the number of variants in the 99% credible set was reduced from 16 (covering 94.8 kb) to 5 (covering 19.6 kb, including a missense variant rs1260326) after trans-ethnic meta-analysis. We also confirmed the previously reported associations of *PKD2L1*, *FADS1/2*, *GCKR*, and *HIF1AN* with palmitoleic acid and of *FADS1/2* and *LPCAT3* with oleic acid in the Chinese-specific GWAS and the trans-ethnic meta-analyses. Pathway-based analyses suggested that the identified loci were in unsaturated FA metabolism and signaling pathways. Our findings provide novel insight into the genetic basis relevant to MUFA metabolism and biology.

MUFAs are nonessential unsaturated FAs with one double bond in the carbon chain, mainly including palmitoleic acid (16:1n-7), vaccenic acid (18:1n-7), oleic acid (18:1n-9), gondoic acid (20:1n-9), erucic acid (22:1n-9), and nervonic acid (24:1n-9). Oleic acid, the most abundant MUFA in lipids (1), is the predominant dietary MUFA (rich in plant oils, such as olive, canola, hazelnut, almond, and rapeseed, and in animal-derived fats, such as lard, tallow, and butter) (1, 2). Oleic and palmitoleic acid can be synthesized through de novo lipogenesis (DNL) by Δ-9 desaturation of stearic and palmitic acid, respectively, in liver and adipose tissue (3, 4). Vaccenic, gondoic, erucic, and nervonic acids are elongation products of palmitoleic or oleic acid through endogenous synthesis (1).

MUFAs are important components of cell membranes and serve as energy sources through β-oxidation in the mitochondria (for example, in the skeletal muscle during exercise) (5). Previous animal studies and clinical trials have suggested that palmitoleic and oleic acid play a role in lipid and glucose regulation (6–9). In addition, epidemiological studies indicated that elevated levels of specific plasma and erythrocyte membrane MUFA levels (palmitoleic, vaccenic, erucic, and nervonic acids) were associated with increased risk of T2D (10), metabolic abnormalities (11), and CVD (12–14) in European populations, and studies in Chinese Hans also observed that higher levels of erythrocyte palmitoleic and oleic acid levels were associated with increased risk of metabolic syndrome (MS) and T2D (15, 16). Therefore, these MUFAs are of great importance to cardiometabolic diseases.

Recently, genome-wide association studies (GWASs) have identified seven loci (*FADS1/2*, *PKD2L1*, *HIF1AN*, *GCKR*, *2p13*,* LPCAT3*, and *TRIM58*) associated with plasma and erythrocyte palmitoleic acid and/or oleic acid levels in populations of European ancestry (17, 18). However, these loci have not been replicated in other ethnic groups with different genetic architecture and dietary intake (19). More importantly, the potential functional variants at these loci and the genetic variants that affect circulating levels of vaccenic, gondoic, erucic, and nervonic acid remain unknown. Therefore, we first performed ethnic-specific GWAS meta-analyses in populations consisting of 3,521 Chinese individuals from two cohorts and of 12,020 European individuals from eight cohorts, respectively, and then combined the GWAS data from these two ethnic groups by trans-ethnic meta-analysis. We further conducted trans-ethnic fine-mapping of the identified loci by construction of 99% credible sets.

## MATERIALS AND METHODS

### Study cohorts

Chinese-specific GWAS meta-analysis included data on 3,521 Chinese-ancestry participants from two cohorts: the Nutrition and Health of Aging Population in China (NHAPC) (20) and the Multi-Ethnic Study of Atherosclerosis (MESA) (21). European-specific GWAS meta-analysis included data on 12,020 European-ancestry participants from eight cohorts: the Atherosclerosis Risk in Communities study (ARIC) (22), the Coronary Artery Risk Development in Young Adults study (CARDIA) (23), the Cardiovascular Health Study (CHS) (24), the Genetics of Lipid Lowering Drugs and Diet Network (GOLDN) (25), the Nurses’ Health Study (NHS) (26), the Health Professionals Follow-Up Study (HPFS) (27), the Invecchiare in Chianti Study (InCHIANTI) (28), and MESA. Summary statistics of the two ethnic-specific GWAS meta-analyses were combined in the trans-ethnic meta-analysis. Written informed consent was obtained from all participants, and each study was approved by local ethics committees. Detailed descriptions of each cohort are provided in the supplemental Methods.

### FA measurements

For ARIC, CARDIA, CHS, and MESA, fasting plasma phospholipids were isolated by TLC, and FAs were subsequently quantified by gas chromatography. For InCHIANTI, fasting FAs were measured in total plasma by gas chromatography. For GOLDN, NHS, HPFS, and NHAPC, fasting erythrocyte FAs were measured by gas chromatography or gas-liquid chromatography. FAs were identified for each study, and concentration of each FA was expressed as percentage of total FAs. Detailed methods of MUFA measurement in each cohort are provided in supplemental Methods. Ethnic-specific GWAS meta-analyses were performed for associations with palmitoleic, vaccenic, oleic, gondoic, erucic, and nervonic acids in Chinese populations, and for associations with vaccenic, gondoic, erucic, and nervonic acids in European populations (**Table 1**).

**TABLE 1.** Basic characteristics of study populations

Study	Ethnicity	N	Age, Year	Men, %	Sample Type	FAs (%)					
						16:1n-7	18:1n-7	18:1n-9	20:1n-9	22:1n-9	24:1n-9
Chinese populations											
NHAPC	Chinese	2,865	58.6 (6.0)	43.2	RBC	0.41 (0.20)	1.02 (0.17)	11.1(1.38)	0.29 (0.15)	0.16 (0.18)	4.21 (1.78)
MESA Chinese	Chinese-American	656	62.5 (10.3)	48.6	Plasma PL	0.44 (0.16)	1.50 (0.25)	7.30(1.10)	0.14 (0.05)	NA	0.80 (0.44)
European populations											
ARIC	European-American	3,269	53.8 (5.6)	48.7	Plasma PL	—	NA	—	0.12 (0.03)	NI	0.57 (0.17)
CARDIA	European-American	1,507	45.8 (3.4)	46.7	Plasma PL	—	1.24 (0.34)	—	0.12 (0.03)	NA	0.69 (0.33)
CHS	European-American	2,404	75.0 (5.1)	38.4	Plasma PL	—	1.30 (0.20)	—	0.12 (0.03)	0.03 (0.01)	1.92 (0.42)
GOLDN	European-American	1,123	48.2 (16.3)	48.2	RBC	—	NA	—	0.17 (0.13)	NA	NA
NHS	European-American	655	59.9 (6.5)	0	RBC	—	1.20 (0.21)	—	0.18 (0.06)	NA	2.73 (0.71)
HPFS	European-American	1,295	64.3 (8.6)	100	RBC	—	1.08 (0.14)	—	0.19 (0.04)	NA	3.76 (0.79)
InCHIANTI	European-Italian	1,075	68.4 (15.5)	45.1	Plasma	—	1.37 (0.37)	—	0.21 (0.06)	0.78 (0.72)	0.34 (0.24)
MESA White	European-American	692	61.6 (10.4)	47.3	Plasma PL	—	1.34 (0.23)	—	0.13 (0.06)	NA	0.53 (0.30)

Values in the table are mean (SD) except specified otherwise. NA, not available; NI, not included; PL, phospholipids; RBC, red blood cell; —, published GWAS.

### Genotyping and quality control

Illumina 370, 550, and 660W genotyping arrays were used on DNA samples from CHS, InCHIANTI, and NHAPC, respectively, while Affymetrix 6.0 genotyping arrays were used in ARIC, CARDIA, GOLDN, NHS, HPFS, and MESA. SNPs with call rate <95% (ARIC, CARDIA, MESA, NHS, and HPFS) or <97% (CHS, GOLDN, InCHIANTI, and NHAPC) were excluded. SNPs with significant departure from Hardy-Weinberg equilibrium expectations at *P* < 10^−4^ (for NHS and HPFS), *P* < 10^−5^ (for ARIC and CHS), or *P* < 10^−6^ (for CARDIA, GOLDN, InCHIANTI, MESA, and NHAPC) were excluded. SNPs with minor allele frequency <1% were also excluded from these analyses. The software used for imputation included MACH (29) (ARIC, GOLDN, NHS, HPFS, and InCHIANTI), BEAGLE (30) (CARDIA), BIMBAM (31) (CHS), and IMPUTE (32) (MESA and NHAPC). SNPs with imputation information measure <0.5 or imputation quality score (estimated *r*^2^) <0.3 were excluded (33). Methods of genotyping, imputation, and quality control for each cohort are explained in detail in supplemental Methods.

### Chinese- and European-specific GWAS meta-analyses

For each individual FA, GWAS analysis of approximately 2.2 million genotyped and imputed SNPs was performed separately in each cohort. Linear regression models were applied to test associations between SNPs and individual FA levels under an additive genetic model. All analyses were adjusted for age, sex, site of recruitment (as needed), and principal components to account for population admixture. Genomic control corrections were applied to each study before meta-analysis to further minimize potential confounding by population stratification (34). Cohort-specific association results were then combined by using inverse-variance based meta-analysis in METAL software (http://www.sph.umich.edu/csg/abecasis/metal). In Chinese- and European-specific meta-analyses, SNPs that were present in only one study were excluded. The linkage disequilibrium (LD) measures (*r*^2^) were calculated by using the HapMap Phase II data (http://archive.broadinstitute.org/mpg/snap/ldsearchpw.php).

### Trans-ethnic meta-analysis

Trans-ethnic meta-analysis was performed to identify additional novel loci for MUFA levels, to narrow the functional regions represented by the identified association signals, and also to test the heterogeneity of the identified loci across ethnic groups. Association statistics in the Chinese and European populations were combined by using Meta-ANalysis of Trans-ethnic Association studies (MANTRA; using a Bayesian framework) (35). MANTRA allows for heterogeneity among distinct ethnic populations and has increased power and mapping resolution compared with fixed- and random-effects meta-analysis (35). A log_10_[Bayes factor (BF)] ≥6 is considered as significant evidence of an association, and SNPs with posterior probability of heterogeneity (Phet) >0.5 were interpreted as having significant heterogeneity. Trans-ethnic meta-analysis using the fixed-effect model by METAL software was also performed. The 95% and 99% credible sets surrounding the most significant SNP at each locus based on the European-ancestry GWAS meta-analysis and the trans-ethnic meta-analysis of European and Chinese populations were calculated, respectively. The 95% and 99% credible sets at each locus was established by *1*) defining a ±500 kb region surrounding the most significant SNP; *2*) ranking the regional SNPs within this region according to their BF values; and *3*) adding SNPs until the cumulative posterior probabilities of the ranked SNPs achieved the 95% and 99% confidence. Variants in the 99% credible sets were examined for sequence overlap with potential regulatory sites (promoter histone marks, enhancer histone marks, and DNase hypersensitivity) by searching the HaploReg V4.1 database (36). The location of each SNP in the fine-mapping section was extracted from the 1000 Genomes Brower (https://www.ncbi.nlm.nih.gov/variation/tools/1000genomes/).

### *cis*-Expression quantitative trait loci analysis

In the *cis*-expression quantitative trait loci (*cis*-eQTL) analysis, we examined the associations of significant SNPs (**Table 2**) with RNA levels of nearby genes in the public Genotype-Tissue Expression database (GTEx; http://gtexportal.org/home/). Adipose tissue, skeletal muscle, and liver were considered as the most important tissues in FA metabolism (4), and these three tissues were selected in the GTEx browser.

**TABLE 2.** Significant associations observed in the trans-ethnic meta-analysis

FA and Gene	SNP	Chr	Position	EA/NEA	EAF, Chinese/Europeans	Chinese		European		MANTRA		METAL	
						β (SE)	*P*	β (SE)	*P*	BF	Phet	β (SE)	*P*
Novel associations													
18:1n-7													
* PKD2L1/SCD*	rs603424	10	102,065,469	A/G	0.072/0.204	−0.016 (0.009)	0.065	−0.035 (0.004)	5.75 × 10^−17^	14.8	0.090	−0.032 (0.004)	1.65 × 10^−16^
* FADS1/2*	rs174528	11	61,300,075	C/T	0.389/0.368	0.013 (0.005)	3.67 × 10^−3^	0.021 (0.004)	9.90 × 10^−9^	8.07	0.023	0.018 (0.003)	7.22 × 10^−10^
20:1n-9													
* FADS1/2*	rs174601	11	61,379,716	T/C	0.536/0.365	0.008 (0.002)	1.09 × 10^−3^	0.007 (0.001)	3.99 × 10^−38^	43.2	0.007	0.007 (0.001)	1.47 × 10^−45^
* GCKR*	rs780094	2	27,594,741	T/C	0.490/0.412	−0.002 (0.002)	0.450	−0.002 (0.000)	6.31 × 10^−7^	6.22	0.007	−0.002 (0.000)	2.95 × 10^−8^
Previously reported associations													
16:1n-7													
* FADS1/2*	rs102275	11	61,314,379	C/T	0.432/0.329	0.021 (0.005)	1.39 × 10^−5^	0.023 (0.003)*^a^*	2.22 × 10^−12^	14.6	0.006	0.022 (0.003)	2.49 × 10^−16^
* PKD2L1*	rs603424	10	102,065,469	A/G	0.073/0.192	−0.023 (0.009)	6.57 × 10^−3^	−0.032 (0.004)*^a^*	5.32 × 10^−14^	13.0	0.016	−0.030 (0.004)	4.24 × 10^−15^
* GCKR*	rs780093	2	27,596,107	T/C	0.524/0.409	0.017 (0.005)	1.36 × 10^−4^	0.020 (0.003)*^a^*	3.42 × 10^−9^	10.3	0.020	0.019 (0.003)	4.47 × 10^−12^
* HIF1AN*	rs10883511	10	102,289,397	G/A	0.143/0.213	0.026 (0.007)	1.90 × 10^−4^	0.023 (0.004)*^a^*	6.44 × 10^−8^	8.84	0.019	0.024 (0.004)	7.90 × 10^−11^
18:1n-9													
* FADS1/2*	rs102275	11	61,314,379	C/T	0.419/0.329	0.204 (0.032)	2.92 × 10^−10^	0.229 (0.020)*^a^*	1.10 × 10^−31^	38.0	0.061	0.222 (0.017)	1.44 × 10^−39^
* LPCAT3*	rs12579775	12	6,955,432	A/G	0.032/0.094	−0.539 (0.096)	1.86 × 10^−8^	0.024 (0.032)*^a^*	0.451	6.12	1	−0.034 (0.031)	0.282

Chr, chromosome; EA, effect allele; EAF, effect allele frequency; log_10_(BF), log_10_(BF) calculated from MANTRA analyses; NEA, noneffect allele; Phet, Phet between different ethnics.

a These results were previously published in European populations from the CHARGE consortium (18).

### Gene- and pathway-based analysis

For the gene-based analysis, sum of powered score (SPUs) (37) and gene-based association test using extended Simes procedure (GATES) (38) were used, which integrated GWAS summary statistics based on SNP positions and LD information. SNPs were assigned to specific genes by PLINK (http://zzz.bwh.harvard.edu/plink/). For the pathway-based analysis, aSPUpath (37) and GATES-Simes (39) were used. These methods were implemented in the public R package aSPU (https://cran.r-project.org/web/packages/aSPU/). In total, 4,405 and 4,502 genes mapped to 186 Kyoto Encyclopedia of Genes and Genomes pathways were analyzed in Chinese and European populations, respectively. The significant threshold after Bonferroni correction was set as 0.05/(4405 × 6) = 1.87 × 10^−6^ and 0.05/(4502 × 6) = 1.85 × 10^−6^ in Chinese and European populations, respectively, for the gene-based analysis, and 0.05/(186 × 6) = 4.48 × 10^−5^ for the pathway-based analysis.

### Associations of the identified loci with cardiometabolic outcomes

The publicly available GWAS results from CARDIoGRAMplusC4D (http://www.cardiogramplusc4d.org/), DIAGRAM (http://diagram-consortium.org/index.html), and GIANT (http://www.broadinstitute.org/collaboration/giant/index.php/) consortia were used to explore the associations of the significant loci (Table 2) identified in our study with coronary artery disease (CAD), T2D, and BMI. SNPs with *P* < 0.05 were considered to be suggestively significant, and SNPs with *P* < 3.33 × 10^−3^ [0.05/(5 × 3)] were significant.

## RESULTS

### Cohort characteristics

The study sample included 3,521 individuals of Chinese origin from the NHAPC and MESA cohorts and 12,020 individuals of European origin from cohorts including ARIC, CARDIA, CHS, GOLDN, NHS, HPFS, InCHIANTI, and MESA (Table 1). Participants comprised mostly middle-aged to older individuals (mean age across the cohorts ranged from 45.8 to 75.0 years), and approximately 50% of them were male, except for the NHS (female only) and HPFS (male only) cohorts. FA levels were all expressed as the percentage of total FAs (Table 1).

### Novel genetic associations

Three loci (*PKD2L1*, *FADS1/2*, and *GCKR*) showed novel genome-wide significant associations with vaccenic acid and/or gondoic acid in the meta-analysis. Minor allele A of *PKD2L1-*rs603424 was significantly associated with lower vaccenic acid level in the European-specific GWAS meta-analysis (*P* = 5.75 × 10^−17^; Table 2, supplemental Table S1, and supplemental Figs. S1 and S2) and in the trans-ethnic meta-analysis (BF = 14.8, *P* = 1.65 × 10^−16^; Table 2). Minor alleles of *FADS1/2* variants were associated with higher vaccenic and gondoic acid levels in the European-specific GWAS meta-analysis (*P* ≤ 9.90 × 10^−9^; Table 2, supplemental Table S1, and supplemental Figs. S1 and S2) and in the trans-ethnic meta-analysis (BF ≥ 8.07, *P* ≤ 7.22 × 10^−10^; Table 2), and were also confirmed in the Chinese-specific GWAS meta-analysis (*P* ≤ 3.67 × 10^−3^; Table 2). Minor allele T of *GCKR*-rs780094 was associated with a lower gondoic acid level in the trans-ethnic meta-analysis (BF = 6.22, *P* = 2.95 × 10^−8^; Table 2) and showed suggestive significance in the European-specific GWAS meta-analysis (*P* = 6.31 × 10^−7^; Table 2). These novel associations exhibited no evidence of heterogeneity between Chinese and European populations, with consistent association directions and comparable effect sizes (Phet ≤ 0.090; Table 2).

### Previously reported genetic associations

Reported associations of *PKD2L1*, *FADS1/2*, *GCKR*, and *HIF1AN* with palmitoleic acid in European populations (18) were confirmed in the Chinese-specific GWAS meta-analysis (*P* ≤ 6.57 × 10^−3^; Table 2) and in the trans-ethnic meta-analysis (BF ≥ 8.84, *P* ≤ 7.90 × 10^−11^; Table 2). Reported association of *FADS1/2* with oleic acid in European populations (17, 18) reached genome-wide significance in the Chinese-specific GWAS meta-analysis (*P* = 2.92 × 10^−10^; Table 2) and in the trans-ethnic meta-analysis (BF = 38.0, *P* = 1.44 × 10^−39^; Table 2). These loci exhibited no evidence of heterogeneity between Chinese and European populations (Phet ≤ 0.061; Table 2). Association of *LPCAT3* with oleic acid, previously reported in the Framingham Heart Offspring Study (17), reached genome-wide significance in the Chinese-specific GWAS meta-analysis (*P* = 1.86 × 10^−8^; Table 2) and in the trans-ethnic meta-analysis using MANTRA (BF = 6.12; Table 2), but it was not replicated in the European populations from the Cohorts for Heart and Aging Research in Genomic Epidemiology (CHARGE) Consortium (18) (*P* = 0.451; Table 2) or in the trans-ethnic meta-analysis using METAL (*P* = 0.282; Table 2). This locus showed significant heterogeneity between Chinese and European populations (Phet = 1; Table 2). In addition, we failed to replicate the previously reported associations of *2p13* with palmitoleic acid (18) in Chinese populations (*P* = 0.992; supplemental Table S2), and of *TRIM58* with oleic acid (17) in the trans-ethnic meta-analysis (BF = 0.005; supplemental Table S2).

### Fine mapping

Although the SNPs showing the strongest association are not necessarily the functional variants, owing to factors such as sampling variation and LD patterns, it is still reasonable to think that functional variants are among the SNPs interrogated in these loci. By comparing the 99% credible sets calculated on the basis of the European-ancestry meta-analysis and on the basis of the trans-ethnic meta-analysis, we observed improvements in fine-mapping resolution at *FADS1/2* and *GCKR* loci.

The number of SNPs in the 99% credible set at *FADS1/2* locus was reduced from 16 (covering 54.1 kb) to 14 (covering 53.5 kb, from rs174535 located 32.3 kb upstream of *FADS2* to rs174577 in the first intron of *FADS2*), 24 (covering 79.6 kb) to 23 (covering 62.0 kb, from rs174528 located 40.2 kb upstream of *FADS2* to rs174578 in the second intron of *FADS2*), and 15 (covering 54.1 kb) to 12 (covering 53.5 kb, from rs174535 located 32.3 kb upstream of *FADS2* to rs174577 in the first intron of *FADS2*), for association with palmitoleic, vaccenic, and oleic acid, respectively (supplemental Tables S3–S5), and these SNPs show strong LD with each other (*r*^2^ ≥ 0.590 in CEU and *r*^2^ ≥ 0.927 in CHB+JPT). After trans-ethnic meta-analysis, the 12 highly correlated SNPs, including rs174535, rs174545, rs174546, rs102275, rs174536, rs174537, rs174550, rs174547, rs174574, rs174576, rs174577, and rs1535 (*r*^2^ = 1 in CEU and *r*^2^ ≥ 0.927 in CHB+JPT, from rs174535 located 32.3 kb upstream of *FADS2* to rs174577 in the first intron of *FADS2*) in the 99% credible set of oleic acid were shared across the associations with palmitoleic, vaccenic, and oleic acid (supplemental Table S3). We further performed a Fisher’s exact test to evaluate whether the SNPs in the trans-ethnic analysis-based 99% credible sets were enriched for potential regulatory sites (promoter histone marks, enhancer histone marks, and DNase hypersensitivity) when compared with the SNPs in the European-ancestry GWAS-based 99% credible sets. However, no significant enrichment was observed after trans-ethnic meta-analysis (*P* ≥ 0.533). These findings suggested that the improved fine-mapping resolution at *FADS1/2* locus is likely resulted from the increased sample size after trans-ethnic meta-analysis.

The greatest improvement in fine-mapping resolution was observed at the *GCKR* locus, where the number of SNPs in the 99% credible set was reduced from 16 (covering 94.8 kb) on the basis of the European-ancestry meta-analysis to five (covering 19.6 kb, from rs1260326 located in the 15th exon of *GCKR* to rs2911711 located 4.0 kb downstream of *GCKR*) after trans-ethnic meta-analysis. The five SNPs in the 99% credible, in strong LD with each other (*r*^2^ ≥ 0.805 in CEU and *r*^2^ ≥ 0.827 in CHB+JPT), included one missense SNP (rs1260326, p.P446L, located in the 15th exon of *GCKR*), two intronic SNPs (rs780094 and rs780093 located in the 16th and 17th intron of *GCKR*, respectively), and two SNPs in the downstream of *GCKR* (rs1260333 and rs2911711 located 2.1 and 4.0 kb downstream of *GCKR*, respectively) (supplemental Tables S4 and S5).

The 99% credible sets based on the European-ancestry and trans-ethnic meta-analyses at *PKD2L1* locus contained only one SNP (rs603424). Therefore, the posterior probability that rs603424 was functional (or tagged an unobserved functional variant) was greater than 99% for *PKD2L1*. *PKD2L1-*rs603424 is located in the second intron of the *PKD2L1* gene and overlapped with an enhancer histone mark (H3K4me1, H3K27ac and H3K9ac) in the adipose tissue when we searched the Roadmap Epigenomics database (40) using HaploReg V4.1 (supplemental Tables S4 and S5). The previously reported signal *HIF1AN*-rs10883511 for association with palmitoleic acid (18) is located 224 kb away from *PKD2L1*-rs603424, and these two SNPs are independent from each other (*r*^2^ = 0.013 in CEU and *r*^2^ = 0.001 in CHB+JPT). Because *PKD2L1*-rs603424 was the strongest association signal (β = −0.032, *P* = 5.32 × 10^−14^) in the ±500 kb genomic region of *HIF1AN*-rs10883511 (β = 0.023, *P* = 6.44 × 10^−8^), we could not construct credible sets or perform trans-ethnic fine-mapping at *HIF1AN* locus.

### *cis*-eQTL analysis

To gain more insight into the potential functional roles of the genome-wide significant loci (Table 2), we performed *cis*-eQTL analysis by searching the publicly available GTEx database in adipose tissue, skeletal muscle, and liver. Results suggested that the minor allele A of *PKD2L1*-rs603424 was significantly associated with decreased RNA level of *SCD* (*stearoyl-CoA desaturase*; encodes the Δ-9 desaturase in the DNL pathway) in the adipose tissue (*P* ≤ 3.94 × 10^−6^; supplemental Table S6). These results further strengthened our findings from the GWAS meta-analyses that the minor allele A of *PKD2L1*-rs603424 was significantly associated with decreased levels of palmitoleic and vaccenic acids (*P* ≤ 5.32 × 10^−14^; Table 2).

### Gene- and pathway-based analysis

Gene-based analysis improves the statistical power by combining all SNPs in a gene into a gene-based score, which reduces the burden of multiple testing and incorporates multiple independent association signals (41). To identify additional genes and pathways that contribute to circulating MUFA levels and gain insight into the underlying mechanisms, we performed gene- and pathway-based association testing using GWAS summary statistics in Chinese and European populations, respectively. Four genes (*FEN1*,* FADS1*,* FADS2*, and *LPCAT3*; 27 SNPs) for association with oleic acid in the Chinese populations and six genes (*SCD*, *WNT8B*, *NDUFB8*,* FEN1*, *FADS1*, and *FADS2*; 64 SNPs) for association with palmitoleic, vaccenic, oleic, and/or gondoic acid in the European populations reached gene-based significance (*P* ≤ 1.60 × 10^−6^; supplemental Table S7) using both SPU and GATES methods. Because the association signals at *WNT8B* and *NDUFB8* genes for association with palmitoleic acid in the European populations are in strong LD with the reported SNP *HIF1AN*-rs10883511 (18) (*r*^2^ ≥ 0.832), we did not consider them as novel loci. Pathways, including biosynthesis of unsaturated FAs, α-linolenic acid metabolism, glycerophospholipid metabolism, and PPAR signaling pathway, were significantly associated with palmitoleic, vaccenic, oleic, and/or gondoic acid levels in the pathway-based analyses (*P* ≤ 2.28 × 10^−5^; supplemental Table S8).

### Associations of the identified loci with cardiometabolic outcomes

Finally, we searched the public GWAS databases of CARDIoGRAMplusC4D, DIAGRAM, and GIANT Consortia to examine whether the significant loci (Table 2) were associated with cardiometabolic outcomes. We found significant association of *FADS1/2*-rs102275 with T2D (*P* = 0.002; supplemental Table S9) and of *GCKR-*rs780094 with BMI (*P* = 6.98 × 10^−5^; supplemental Table S10). Suggestive evidence was also observed for associations with CAD (*PKD2L1*-rs603424; *P* = 0.024; supplemental Table S11) and T2D (*GCKR-*rs780094; *P* = 0.025; supplemental Table S9).

## DISCUSSION

In this first trans-ethnic meta-analysis of MUFA levels in Chinese and European populations, we identified novel associations of *FADS1/2*, *PKD2L1*, and *GCKR* with vaccenic and/or gondoic acid and replicated the previously reported associations with palmitoleic and/or oleic acid for loci at *FADS1/2*, *PKD2L1*, *GCKR*, *HIF1AN*, and *LPCAT3* in the Chinese-specific GWAS and the trans-ethnic meta-analyses. We also observed substantial improvement in the fine-mapping resolution at the *GCKR* locus after trans-ethnic meta-analysis.

The Δ-9 desaturase, encoded by *SCD*, plays an important role in MUFA metabolism (42). It catalyzes the desaturation reaction from palmitic and stearic acid to palmitoleic and oleic acid, respectively, in the DNL pathway. The present study and the previous GWAS (18) have identified significant associations of *PKD2L1* with palmitoleic and vaccenic acid, which is located near *SCD*. The results of the *cis*-eQTL analysis suggested that the most significant SNP, *PKD2L1*-rs603424, was associated with the RNA level of *SCD* in the adipose tissue (*P* ≤ 3.94 × 10^−6^; supplemental Table S5). Therefore, *PKD2L1*-rs603424 may exert its effect on MUFA levels through regulating *SCD* transcription.

Genetic variants at *FADS1/2* also showed significant associations with MUFA levels, including palmitoleic, vaccenic, oleic, and gondoic acid. *FADS1/2* encode Δ5 and Δ6 desaturases, predominantly involved in the PUFA biosynthesis pathway (43). Recent studies have indicated that Δ6 desaturase can also catalyze palmitic and stearic acid to produce other unsaturated FAs (1, 44). Because palmitic and stearic acid serve as the substrates for MUFA endogenous synthesis, it is possible that *FADS1/2* influences specific MUFA levels through substrate regulation. The exact mechanisms underlying the associations between *FADS1/2* variants and MUFA levels merit further investigation.

*GCKR* encodes glucokinase regulator, a protein that inhibits glucokinase (GCK) activity in liver and pancreas (45). In the present study, substantial improvement of fine-mapping resolution at this locus was observed, and the 99% credible set calculated after trans-ethnic meta-analysis highlighted one missense variant (rs1260326, p.P446L). Functional studies have indicated that *GCKR*-rs1260326 played a central role in regulation of GCK activity in the liver, which consequently influenced glycolytic flux and DNL (46, 47). Therefore, it is likely that rs1260326 is the variant driving the association of *GCKR* with palmitoleic acid through modifying the DNL pathway.

*LPCAT3* encodes lysophosphatidylcholine acyltransferase 3, which is involved in lysophospholipid esterification (48). It was confirmed in the trans-ethnic meta-analysis using MANTRA, but not METAL. The inconsistent trans-ethnic meta-analysis results generated from MANTRA and METAL were mainly due to the different methods implemented in these two software. MANTRA takes account of the expected similarity in allelic effects between the most closely related populations by using a Bayesian partition model and also allows for heterogeneity across ethnic groups (49). In contrast, METAL uses a fixed-effect model that assumes the allelic effect to be the same in all populations. Therefore, MANTRA confers significantly higher power than METAL (50).

In the ethnic-specific GWAS meta-analyses, the magnitude and directions of the identified associations were largely consistent across cohorts (supplemental Table S1 and supplemental Fig. S3). Association analyses further stratified by the measurement methods (erythrocyte and plasma phospholipid FAs) did not materially changed the association results, and no additional erythrocyte- or plasma-specific loci were identified (supplemental Table S1). Association results after excluding the InCHIANTI cohort, which measured MUFA levels in total plasma, also remained largely unchanged. These results suggested that differences in MUFA measurements did not introduce noise in the association results and were similar to the findings of previously published GWAS of FAs (18, 51).

In conclusion, this is the first study to provide evidence for the associations of *FADS1/2*, *PKD2L1*, and *GCKR* with vaccenic and/or gondoic acid levels in the populations of Chinese and European origin. Five previously reported loci (*FADS1/2*, *PKD2L1*, *GCKR*, *HIF1AN*, and *LPCAT3*) for palmitoleic and/or oleic acid were also confirmed. Trans-ethnic fine-mapping highlighted a missense variant (rs1260326) at the *GCKR* locus. Our findings shed light on the genetic basis of MUFA biology and establish the foundation for future genetic and functional investigations.

## Supplementary Material

Supplemental Data
