# Supplementary material for: Discovery and fine-mapping of loci associated with MUFAs through trans-ethnic meta-analysis in Chinese and European populations
Source: J Lipid Res. 2017 Apr 28;58(5):974–81. doi: 10.1194/jlr.P071860 (PMC5408616; doi:10.1194/jlr.P071860)
Supplement: Supplemental Data [file supp_58_5_974__index.html]

Discovery and fine-mapping of loci associated with MUFAs through trans-ethnic meta-analysis in Chinese and European populations — Supplemental Data 

# Discovery and fine-mapping of loci associated with MUFAs through trans-ethnic meta-analysis in Chinese and European populations

## Supplemental Data

- Supplemental Tables S1-S3 and S6-S8 Fig S1-S3 (.docx, 4.8 MB) - Supplemental Tables S1-S3 and S6-S8 Fig S1-S3
- Supplemental Table S4-S5 and S9-S11 (.xlsx, 31 KB) - Supplemental Table S4-S5 and S9-S11
- Supplemental methods (.docx, 56 KB) - Supplemental methods
