# Supplementary material for: Discovery and fine-mapping of loci associated with MUFAs through trans-ethnic meta-analysis in Chinese and European populations
Source: J Lipid Res. 2017 Apr 28;58(5):974–81. doi: 10.1194/jlr.P071860 (PMC5408616; doi:10.1194/jlr.P071860)

Supplemental Table S1. Ethnic-specific and stratified analysis GWAS meta-analysis

| Fatty acid | Gene | SNP | Chr | Position | EA/NEA | EAF | beta(SE) | *P* | *P _for heterogeneity_* |
| --- | --- | --- | --- | --- | --- | --- | --- | --- | --- |
| Chinese-specific GWAS meta-analysis | | | | | | | | | |
| 18:1n-9 | *FADS1/2* | rs174601 | 11 | 61379716 | T/C | 0.423 | 0.212(0.034) | 2.56×10^-10^ | 0.872 |
| Red blood cell MUFA-specific GWAS in Chinese | | | | | | | | | |
| 18:1n-9 | *FADS1/2* | rs174601 | 11 | 61379716 | T/C | 0.367 | 0.209(0.038) | 4.96×10^-8^ | NA |
| Plasma phospholipid MUFA-specific GWAS in Chinese | | | | | | | | | |
| 18:1n-9 | *FADS1/2* | rs174601 | 11 | 61379716 | T/C | 0.590 | 0.222(0.066) | 8.08×10^-4^ | NA |
| European-specific GWAS meta-analysis | | | | | | | | | |
| 18:1n-7 | *PKD2L1* | rs603424 | 10 | 102065469 | A/G | 0.204 | -0.035(0.004) | 5.75×10^-17^ | 6.26×10^-3^ |
|  | *FADS1/2* | rs174528 | 11 | 61300075 | C/T | 0.368 | 0.021(0.004) | 9.90×10^-9^ | 0.996 |
| 20:1n-9 | *FADS1/2* | rs174528 | 11 | 61300075 | C/T | 0.364 | 0.007(0.001) | 2.91×10^-46^ | 4.42×10^-3^ |
| Red blood cell MUFA-specific GWAS in Europeans | | | | | | | | | |
| 18:1n-7 | *PKD2L1* | rs603424 | 10 | 102065469 | A/G | 0.193 | -0.022(0.006) | 3.29×10^-4^ | 0.091 |
|  | *FADS1/2* | rs174528 | 11 | 61300075 | C/T | 0.372 | 0.021(0.005) | 1.64×10^-4^ | 0.923 |
| 20:1n-9 | *FADS1/2* | rs174528 | 11 | 61300075 | C/T | 0.372 | 0.009(0.001) | 1.96×10^-9^ | 0.964 |
| Plasma phospholipid MUFA-specific GWAS in Europeans | | | | | | | | | |
| 18:1n-7 | *PKD2L1* | rs603424 | 10 | 102065469 | A/G | 0.213 | -0.047(0.006) | 4.43×10^-16^ | 0.214 |
|  | *FADS1/2* | rs174528 | 11 | 61300075 | C/T | 0.364 | 0.021(0.005) | 1.55×10^-5^ | 0.950 |
| 20:1n-9 | *FADS1/2* | rs174528 | 11 | 61300075 | C/T | 0.363 | 0.007(0.001) | 8.03×10^-39^ | 9.43×10^-4^ |
| Excluding InCHIANTI in European-specific GWAS meta-analysis | | | | | | | | | |
| 18:1n-7 | *PKD2L1* | rs603424 | 10 | 102065469 | A/G | 0.199 | -0.037(0.004) | 3.77×10^-17^ | 5.63×10^-3^ |
|  | *FADS1/2* | rs174528 | 11 | 61300075 | C/T | 0.369 | 0.021(0.004) | 2.03×10^-8^ | 0.985 |
| 20:1n-9 | *FADS1/2* | rs174528 | 11 | 61300075 | C/T | 0.365 | 0.007(0.001) | 6.55×10^-42^ | 0.066 |

SNP, single nucleotide polymorphism; Chr, chromosome; EA, effect allele; NEA, non-effect allele; EAF, effect allele frequency.

Supplemental Table S2. Failed replication of previously reported loci *2p13* and *TRIM58*

| Fatty acid | Gene | SNP | Chr | Position | EA | EAF  (Chinese/Europeans) | Chinese | | Europeans | | MANTRA | | METAL | |
| --- | --- | --- | --- | --- | --- | --- | --- | --- | --- | --- | --- | --- | --- | --- |
|  |  |  |  |  |  |  | beta (SE) | *P* | beta (SE) | *P* | BF | Phet | beta (SE) | *P* |
| 16:1n-7 | *2p13* | rs6722456 | 2 | 134245561 | A | 0.183/0.023 | 0.000(0.006) | 0.992 | -0.048(0.009) | 3.65×10^-8^ | 4.47 | 0.996 | -0.016(0.005) | 1.97×10^-3^ |
| 18:1-9 | *TRIM58* | rs3811444 | 1 | 246106074 | T | 0.227/0.347 | -0.096(0.038) | 0.012 | -0.003(0.030) | 0.914*^1^* | 0.005 | 0.205 | -0.039(0.024) | 0.100 |

SNP, single nucleotide polymorphism; Chr, chromosome; EA, effect allele; EAF, effect allele frequency.

*^1^* The association of *TRIM58-*rs3811444 with oleic acid was reported by the Framingham Heart Offspring Study (17) and the association result in the table was from the European studies within the CHARGE Consortium (18).

Supplemental Table S3. Fine mapping of MUFA loci with MANTRA

| Chr | Lead SNP | European and Chinese ancestry | | | | | European ancestry only | | | | |
| --- | --- | --- | --- | --- | --- | --- | --- | --- | --- | --- | --- |
|  |  | BF | 95% set | 95% interval | 99% set | 99% interval | BF | 95% set | 95% interval | 99% set | 99% interval |
| 16:1n-7 |  |  |  |  |  |  |  |  |  |  |  |
| 11 | rs102275 | 14.6 | 9 | 48986 | 14 | 53458 | 10.2 | 13 | 53458 | 16 | 54143 |
| 10 | rs603424 | 13.0 | 1 | 0 | 1 | 0 | 11.4 | 1 | 0 | 1 | 0 |
| 2 | rs780093 | 10.3 | 3 | 9309 | 5 | 19606 | 7.21 | 9 | 55248 | 16 | 94775 |
| 18:1n-7 |  |  |  |  |  |  |  |  |  |  |  |
| 10 | rs603424 | 14.8 | 1 | 0 | 1 | 0 | 14.9 | 1 | 0 | 1 | 0 |
| 11 | rs174528/rs174548 | 8.07 | 20 | 62000 | 23 | 62000 | 6.06 | 20 | 66251 | 24 | 79641 |
| 18:1n-9 |  |  |  |  |  |  |  |  |  |  |  |
| 11 | rs102275 | 38.0 | 9 | 48986 | 12 | 53458 | 29.3 | 12 | 54143 | 15 | 54143 |
| 20:1n-9 |  |  |  |  |  |  |  |  |  |  |  |
| 11 | rs174601 | 43.2 | 2 | 79641 | 7 | 79641 | 40.9 | 1 | 0 | 5 | 79641 |

Chr, chromosome; BF, log_10_(Bayes factor).

Supplemental Table S6. Association of SNPs for MUFA levels with mRNA expression levels

| SNP | Gene | Chr | Correlated gene | *P* | Tissue ^b^ |
| --- | --- | --- | --- | --- | --- |
| rs102275 | *C11orf10* | 11 | *FADS1* | 2.08×10^-6^ | Skeletal muscle |
| rs102275 | *C11orf10* | 11 | *FADS2* | 9.07×10^-8^ | Skeletal muscle |
| rs174528 | *C11orf9* | 11 | *FADS2* | 3.25×10^-7^ | Skeletal muscle |
| rs174528 | *C11orf9* | 11 | *TMEM258* | 7.24×10^-6^ | Skeletal muscle |
| rs174601 | *FADS2* | 11 | *FADS1* | 8.52×10^-8^ | Skeletal muscle |
| rs603424 | *PKD2L1* | 10 | *SCD* | 3.94×10^-6^ | Subcutaneous adipose |
| rs603424 | *PKD2L1* | 10 | *SCD* | 1.06×10^-6^ | Visceral adipose |
| rs780093 | *GCGR* | 2 | *SNX17* | 3.81×10^-10^ | Skeletal muscle |
| rs780094 | *GCGR* | 2 | *SNX17* | 2.71×10^-10^ | Skeletal muscle |
| rs10883511 | *HIF1AN* | 10 | *SEC31B* | 5.15×10^-31^ | Subcutaneous adipose |
| rs10883511 | *HIF1AN* | 10 | *SEC31B* | 5.38×10^-18^ | Visceral adipose |
| rs10883511 | *HIF1AN* | 10 | *HIF1AN* | 1.12×10^-8^ | Skeletal muscle |
| rs10883511 | *HIF1AN* | 10 | *SEC31B* | 3.21×10^-12^ | Skeletal muscle |
| rs12579775 | *EMG1* | 12 | *LPCAT3* | 6.99×10^-8^ | Skeletal muscle |

SNP, single nucleotide polymorphism; Chr, chromosome.

Supplemental Table S7. Significant genes associated with MUFA levels in Chinese and European populations using gene-based testing

| Gene ^1^ | Chr | Start position  (Build 36) | End position  (Build 36) | Ancestry | Number  of SNPs | *P* values | |
| --- | --- | --- | --- | --- | --- | --- | --- |
|  |  |  |  |  |  | SPU | GATES |
| 16:1n-7 | | | | | | | |
| *STX19* | 3 | 95215904 | 95230144 | Chinese | 4 | <0.0001 | 2.73×10^-4^ |
| *PSTK* | 10 | 124729545 | 124739896 | Chinese | 10 | <0.0001 | 1.95×10^-5^ |
| *ACADSB* | 10 | 124758418 | 124807796 | Chinese | 35 | <0.0001 | 2.19×10^-5^ |
| *FEN1* | 11 | 61316725 | 61321286 | Chinese | 1 | <0.0001 | 1.72×10^-5^ |
| *FADS1* | 11 | 61323676 | 61340886 | Chinese | 8 | <0.0001 | 1.84×10^-5^ |
| *FADS2* | 11 | 61352288 | 61391401 | Chinese | 15 | <0.0001 | 2.01×10^-4^ |
| *CAD* | 2 | 27293761 | 27320158 | European | 23 | <0.0001 | 5.01×10^-4^ |
| *ADCY1* | 7 | 45580649 | 45729239 | European | 75 | <0.0001 | 3.29×10^-3^ |
| ***SCD*** | **10** | **102096761** | **102114578** | **European** | **13** | **<0.0001** | **1.60×10^-6^** |
| ***WNT8B*** | **10** | **102212801** | **102233389** | **European** | **11** | **<0.0001** | **1.72×10^-7^** |
| ***NDUFB8*** | **10** | **102273486** | **102279626** | **European** | **3** | **<0.0001** | **1.85×10^-8^** |
| ***FEN1*** | **11** | **61316725** | **61321286** | **European** | **3** | **<0.0001** | **6.40×10^-10^** |
| ***FADS1*** | **11** | **61323676** | **61340886** | **European** | **8** | **<0.0001** | **1.44×10^-11^** |
| ***FADS2*** | **11** | **61352288** | **61391401** | **European** | **22** | **<0.0001** | **6.92×10^-11^** |
| *HERC2* | 15 | 26029782 | 26240890 | European | 37 | <0.0001 | 2.53×10^-5^ |
| 18:1n-7 | | | | | | | |
| *SERPING1* | 11 | 57121602 | 57138902 | Chinese | 9 | <0.0001 | 1.80×10^-4^ |
| *C6* | 5 | 41178092 | 41297297 | European | 115 | <0.0001 | 6.60×10^-5^ |
| *DUSP4* | 8 | 29249536 | 29264104 | European | 8 | <0.0001 | 1.74×10^-4^ |
| *CUL2* | 10 | 35338811 | 35419300 | European | 50 | <0.0001 | 5.36×10^-5^ |
| ***SCD*** | **10** | **102096761** | **102114578** | **European** | **13** | **<0.0001** | **3.04×10^-10^** |
| *WNT8B* | 10 | 102212801 | 102233389 | European | 11 | <0.0001 | 8.34×10^-6^ |
| *NDUFB8* | 10 | 102273486 | 102279626 | European | 3 | <0.0001 | 4.09×10^-6^ |
| ***FEN1*** | **11** | **61316725** | **61321286** | **European** | **3** | **<0.0001** | **4.81×10^-7^** |
| ***FADS1*** | **11** | **61323676** | **61340886** | **European** | **8** | **<0.0001** | **8.74×10^-8^** |
| ***FADS2*** | **11** | **61352288** | **61391401** | **European** | **26** | **<0.0001** | **1.34×10^-6^** |
| *CCL5* | 17 | 31222608 | 31231490 | European | 6 | <0.0001 | 8.15×10^-5^ |
| 18:1n-9 | | | | | | | |
| ***FEN1*** | **11** | **61316725** | **61321286** | **Chinese** | **1** | **<0.0001** | **5.94×10^-10^** |
| ***FADS1*** | **11** | **61323676** | **61340886** | **Chinese** | **8** | **<0.0001** | **7.96×10^-10^** |
| ***FADS2*** | **11** | **61352288** | **61391401** | **Chinese** | **15** | **<0.0001** | **1.05×10^-9^** |
| ***LPCAT3*** | **12** | **6955607** | **6996103** | **Chinese** | **3** | **<0.0001** | **4.19×10^-7^** |
| *BLM* | 15 | 89061582 | 89159690 | Chinese | 54 | <0.0001 | 1.85×10^-3^ |
| *ITGB6* | 2 | 160666478 | 160764836 | European | 83 | <0.0001 | 7.24×10^-4^ |
| *PPT2* | 6 | 32229278 | 32239430 | European | 2 | <0.0001 | 4.61×10^-6^ |
| *AGPAT1* | 6 | 32243966 | 32253820 | European | 9 | <0.0001 | 1.26×10^-5^ |
| ***FEN1*** | **11** | **61316725** | **61321286** | **European** | **3** | **<0.0001** | **1.49×10^-28^** |
| ***FADS1*** | **11** | **61323676** | **61340886** | **European** | **8** | **<0.0001** | **3.41×10^-31^** |
| ***FADS2*** | **11** | **61352288** | **61391401** | **European** | **22** | **<0.0001** | **2.71×10^-30^** |
| 20:1n-9 | | | | | | | |
| *NPFFR2* | 4 | 73116384 | 73232642 | European | 23 | <0.0001 | 4.86×10^-5^ |
| ***FEN1*** | **11** | **61316725** | **61321286** | **European** | **3** | **<0.0001** | **2.04×10^-38^** |
| ***FADS1*** | **11** | **61323676** | **61340886** | **European** | **8** | **<0.0001** | **7.65×10^-43^** |
| ***FADS2*** | **11** | **61352288** | **61391401** | **European** | **26** | **<0.0001** | **1.59×10^-41^** |
| 22:1n-9 | | | | | | | |
| *FEN1* | 11 | 61316725 | 61321286 | European | 3 | <0.0001 | 2.40×10^-4^ |
| *FADS1* | 11 | 61323676 | 61340886 | European | 8 | <0.0001 | 3.16×10^-4^ |
| *APOA5* | 11 | 116165295 | 116167794 | European | 2 | <0.0001 | 2.94×10^-5^ |
| *PFAS* | 17 | 8093361 | 8114528 | European | 7 | <0.0001 | 7.45×10^-5^ |
| 24:1n-9 | | | | | | | |
| *PPP3CA* | 4 | 102163609 | 102487376 | Chinese | 176 | <0.0001 | 5.60×10^-4^ |
| *ULBP3* | 6 | 150427435 | 150431895 | Chinese | 3 | <0.0001 | 7.49×10^-5^ |
| *MAGI1* | 3 | 65314945 | 65999549 | European | 622 | <0.0001 | 2.16×10^-4^ |
| *PPA2* | 4 | 106509682 | 106614676 | European | 122 | <0.0001 | 3.06×10^-5^ |
| *APOA5* | 11 | 116165295 | 116167794 | European | 2 | <0.0001 | 1.04×10^-3^ |
| *MAP3K14* | 17 | 40696270 | 40750197 | European | 27 | <0.0001 | 2.17×10^-3^ |
| *PPP5C* | 19 | 51542133 | 51585945 | European | 31 | <0.0001 | 3.81×10^-4^ |

Chr, chromosome; SNP, single nucleotide polymorphism; SPU, sum of powered score; GATES, gene-based association test using extended Simes procedure.

*^1^* Genes with significant *P* values after Bonferroni correction in both SPU and GATES methods were presented in bold.

Supplemental Table S8. Significant pathways associated with MUFA levels in Chinese and European populations using pathway-based testing

| KEGG code ^1^ | Pathway description | Number  of genes | Number  of SNPs | Contributing  genes *^2^* | Ancestry | *P* values | |
| --- | --- | --- | --- | --- | --- | --- | --- |
|  |  |  |  |  |  | aSPUpath | GATES-Simes |
| 16:1n-7 | | | | | | | |
| hsa00534 | Glycosaminoglycan biosynthesis - heparan sulfate | 23 | 2036 | *EXTL3* | Chinese | 0 | 0.065 |
| hsa00592 | α-Linolenic acid metabolism | 17 | 366 | *FADS2* | Chinese | 0 | 3.42×10^-3^ |
| hsa00970 | Aminoacyl-tRNA biosynthesis | 41 | 1425 | *PSTK, IARS, SARS* | Chinese | 0 | 7.99×10^-4^ |
| hsa01040 | Biosynthesis of unsaturated fatty acids | 19 | 748 | *FADS1, FADS2* | Chinese | 0 | 3.50×10^-4^ |
| hsa03030 | DNA replication | 31 | 799 | *FEN1, SSBP1* | Chinese | 0 | 5.33×10^-4^ |
| hsa03410 | Base excision repair | 26 | 454 | *FEN1* | Chinese | 0 | 4.47×10^-4^ |
| hsa03450 | Non-homologous end-joining | 12 | 496 | *FEN1* | Chinese | <0.00001 | 2.06×10^-4^ |
| **hsa00190** | **Oxidative phosphorylation** | **97** | **1818** | ***NDUFB8, ATP6V1E1, NDUFAB1, NDUFC2, NDUFS5*** | **European** | **0** | **1.81×10^-6^** |
| **hsa00592** | **α-Linolenic acid metabolism** | **17** | **430** | ***FADS2*** | **European** | **0** | **1.18×10^-9^** |
| **hsa01040** | **Biosynthesis of unsaturated fatty acids** | **20** | **906** | ***FADS1, FADS2, SCD, PECR*** | **European** | **0** | **2.87×10^-10^** |
| **hsa03030** | **DNA replication** | **33** | **839** | ***FEN1, MCM6*** | **European** | **0** | **2.11×10^-8^** |
| **hsa03320** | **PPAR signaling pathway** | **61** | **2067** | ***FADS2, SCD, SLC27A6*** | **European** | **0** | **4.22×10^-9^** |
| **hsa03410** | **Base excision repair** | **28** | **584** | ***FEN1*** | **European** | **0** | **1.79×10^-8^** |
| **hsa03450** | **Non-homologous end-joining** | **13** | **542** | ***FEN1*** | **European** | **0** | **8.32×10^-9^** |
| **hsa04310** | **Wnt signaling pathway** | **137** | **6883** | ***WNT8B, CER1, CTBP2, RHOA, SMAD2*** | **European** | **0** | **2.35×10^-5^** |
| **hsa04340** | **Hedgehog signaling pathway** | **53** | **1970** | ***WNT8B, DHH, SUFU*** | **European** | **0** | **9.11×10^-6^** |
| **hsa04916** | **Melanogenesis** | **91** | **5414** | ***WNT8B, ADCY1, KRAS*** | **European** | **0** | **1.56×10^-5^** |
| **hsa05010** | **Alzheimer’s disease** | **139** | **8325** | ***NDUFB8, NDUFAB1, GRIN1, NDUFS5, NDUFC2, BID, CALML6, CHP2, APAF1, NDUFV3*** | **European** | **0** | **2.57×10^-6^** |
| **hsa05012** | **Parkinson’s disease** | **92** | **3316** | ***NDUFB8, NDUFAB1, NDUFC2, NDUFS5, VDAC1, APAF1, NDUFV3*** | **European** | **0** | **1.70×10^-6^** |
| **hsa05016** | **Huntington's disease** | **150** | **6063** | ***NDUFB8, NDUFAB1, CLTB, GRIN1, NDUFS5, NDUFC2, IFT57, VDAC1, POLR2K, AP2S1, APAF1, NDUFV3*** | **European** | **0** | **2.78×10^-6^** |
| hsa05200 | Pathways in cancer | 294 | 19319 | *WNT8B, APPL1, IGF1, KRAS, PIK3R5, FASLG, SUFU, CCDC6, SMAD2, FGF21, CTBP2, RHOA, BID, CDC42, TCEB2, FGF5, IGF1R, NFKBIA, CHUK, WNT4* | European | 0 | 5.05×10^-5^ |
| **hsa05217** | **Basal cell carcinoma** | **49** | **1593** | ***WNT8B, SUFU*** | **European** | **0** | **8.42×10^-6^** |
| 18:1n-7 | | | | | | | |
| hsa00190 | Oxidative phosphorylation | 100 | 1832 | *NDUFB8, COX7A1, ATP5C1, NDUFB9, ATP5B* | European | 0 | 4.09×10^-4^ |
| **hsa00592** | **α-Linolenic acid metabolism** | **17** | **440** | ***FADS2*** | **European** | **0** | **2.28×10^-5^** |
| **hsa01040** | **Biosynthesis of unsaturated fatty acids** | **20** | **916** | ***FADS1, FADS2, SCD, ACOT4*** | **European** | **0** | **6.07×10^-9^** |
| **hsa03030** | **DNA replication** | **33** | **860** | ***FEN1, DNA2, RNASEH2A*** | **European** | **0** | **1.59×10^-5^** |
| **hsa03320** | **PPAR signaling pathway** | **61** | **2096** | ***FADS2, SCD*** | **European** | **0** | **1.85×10^-8^** |
| hsa03410 | Base excision repair | 28 | 592 | *FEN1* | European | 0.003 | 1.35×10^-5^ |
| **hsa03450** | **Non-homologous end-joining** | **13** | **552** | ***FEN1, LIG4*** | **European** | **0** | **6.25×10^-6^** |
| hsa04310 | Wnt signaling pathway | 137 | 7028 | *WNT8B, PPP3CB, NFAT5, DAAM2, WNT2B, MAPK8, MAPK9, TP53, GSK3B, TBL1XR1, WNT5B* | European | 0 | 1.14×10^-3^ |
| hsa04320 | Dorso-ventral axis formation | 24 | 1839 | *ETV6, ETS2, NOTCH1* | European | 0 | 8.61×10^-4^ |
| hsa04340 | Hedgehog signaling pathway | 53 | 2013 | *WNT8B, BMP2, WNT2B, GSK3B, ZIC2* | European | 0 | 4.42×10^-4^ |
| hsa05010 | Alzheimer’s disease | 140 | 8430 | *NDUFB8, COX7A1, PPP3CB, NDUFB9, ATP5C1, APP, GSK3B, ATP5B, ITPR1, EIF2AK3* | European | 0 | 5.73×10^-4^ |
| hsa05217 | Basal cell carcinoma | 49 | 1635 | *WNT8B, BMP2, WNT2B, TP53, GSK3B* | European | 0 | 4.09×10^-4^ |
| 18:1n-9 | | | | | | | |
| **hsa00564** | **Glycerophospholipid metabolism** | **68** | **3111** | ***LPCAT3, CHPT1, LYPLA1, AGPAT2, GPD1*** | **Chinese** | **0** | **2.85×10^-5^** |
| **hsa00592** | **α-Linolenic acid metabolism** | **17** | **366** | ***FADS2*** | **Chinese** | **0** | **1.79×10^-8^** |
| **hsa01040** | **Biosynthesis of unsaturated fatty acids** | **19** | **748** | ***FADS1, FADS2, ACAA1*** | **Chinese** | **0** | **9.98×10^-9^** |
| **hsa03030** | **DNA replication** | **31** | **799** | ***FEN1, POLD1*** | **Chinese** | **0** | **1.84×10^-8^** |
| **hsa03320** | **PPAR signaling pathway** | **61** | **1740** | ***FADS2, APOA5, ACADM, SCP2, ACOX2, ACAA1, ACSL6*** | **Chinese** | **0** | **6.41×10^-8^** |
| **hsa03410** | **Base excision repair** | **26** | **454** | ***FEN1, NEIL2, POLD1*** | **Chinese** | **0** | **1.55×10^-8^** |
| **hsa03450** | **Non-homologous end-joining** | **12** | **496** | ***FEN1*** | **Chinese** | **0** | **7.13×10^-9^** |
| **hsa00592** | **α-Linolenic acid metabolism** | **17** | **430** | ***FADS2, PLA2G5, PLA2G2E*** | **European** | **0** | **4.61×10^-29^** |
| **hsa01040** | **Biosynthesis of unsaturated fatty acids** | **20** | **906** | ***FADS1, FADS2, PECR*** | **European** | **0** | **6.81×10^-30^** |
| **hsa03030** | **DNA replication** | **33** | **839** | ***FEN1*** | **European** | **0** | **4.92×10^-27^** |
| **hsa03320** | **PPAR signaling pathway** | **61** | **2067** | ***FADS2, APOA5, ACADL, ACSL3, EHHADH, CPT1A, CD36*** | **European** | **0** | **1.65×10^-28^** |
| **hsa03410** | **Base excision repair** | **28** | **584** | ***FEN1*** | **European** | **0** | **4.18×10^-27^** |
| **hsa03450** | **Non-homologous end-joining** | **13** | **542** | ***FEN1*** | **European** | **0** | **1.94×10^-27^** |
| hsa04142 | Lysosome | 111 | 3055 | *PPT2, CLN3, CTSD, HGSNAT, CLTB* | European | 0 | 5.12×10^-4^ |
| 20:1n-9 | | | | | | | |
| hsa01040 | Biosynthesis of unsaturated fatty acids | 19 | 748 | *FADS1, FADS2* | Chinese | 0 | 6.66×10^-3^ |
| **hsa00592** | **α-Linolenic acid metabolism** | **17** | **450** | ***FADS2*** | **European** | **0** | **2.71×10^-40^** |
| **hsa01040** | **Biosynthesis of unsaturated fatty acids** | **20** | **922** | ***FADS1, FADS2, SCD, ACOT4*** | **European** | **0** | **1.53×10^-41^** |
| **hsa03030** | **DNA replication** | **33** | **885** | ***FEN1, POLE2, POLD1, SSBP1*** | **European** | **0** | **6.73×10^-37^** |
| **hsa03320** | **PPAR signaling pathway** | **61** | **2121** | ***FADS2, SCD, FABP7, LPL, HMGCS2, NR1H3*** | **European** | **0** | **9.72×10^-40^** |
| **hsa03410** | **Base excision repair** | **28** | **608** | ***FEN1, POLE2, POLE1*** | **European** | **0** | **5.71×10^-37^** |
| **hsa03450** | **Non-homologous end-joining** | **13** | **558** | ***FEN1*** | **European** | **0** | **2.65×10^-37^** |

KEGG, Kyoto Encyclopedia of Genes and Genomes; aSPUpath, extended pathway analysis using SPU; GATES-Simes, gene-based association test using extended Simes procedure.

*^1^* Pathways with significant *P* values after Bonferroni correction in both aSPU and GATES-Simes methods were presented in bold.

*^2^* Genes with *P* ≤ 0.05 using SPU and GATES methods were selected as contributing genes.

Supplemental Figure S1. Manhattan plots of genome-wide significant associations with MUFAs. (A) 18:1n-9 in Chinese; (B) 18:1n-7 in Europeans; (C) 20:1n-9 in Europeans. Associations are demonstrated by chromosome location and –log_10_ (*P* value) in Chinese and European populations. Genes of interest in each locus with SNPs that reached genome-wide significance are shown.

(A)


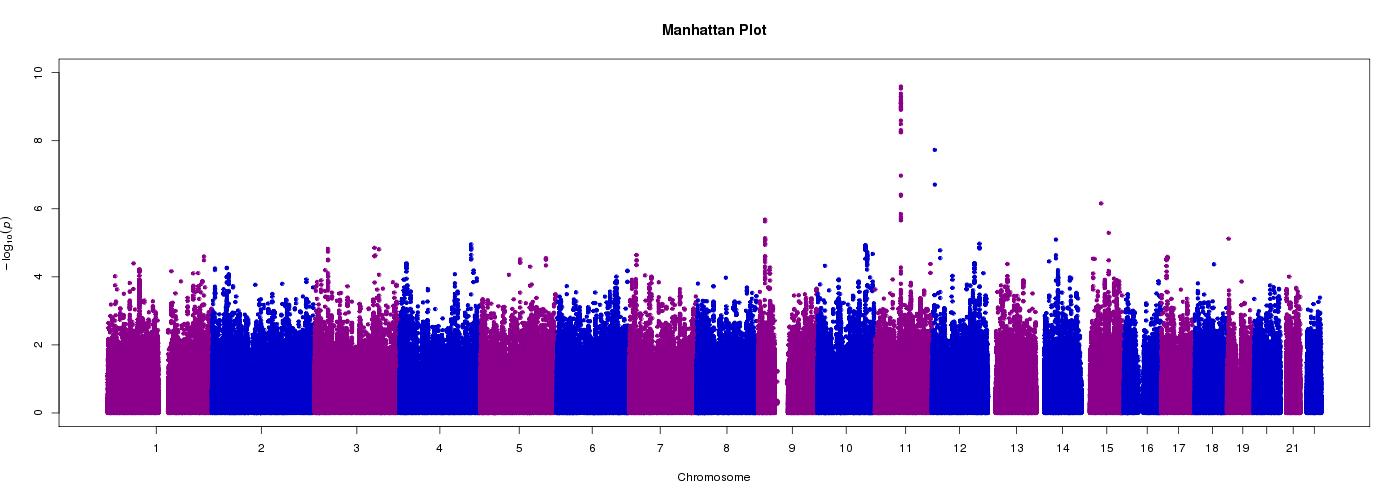


*LPCAT3*

*FADS1/2*

(B)


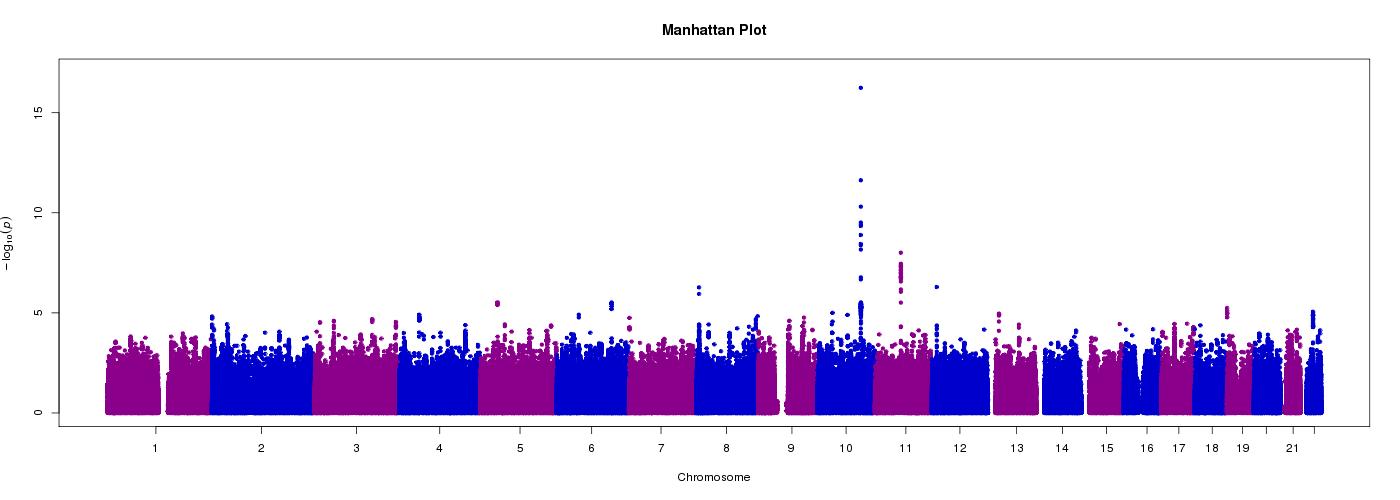


*PKD2L1*

*FADS1/2*

(C)


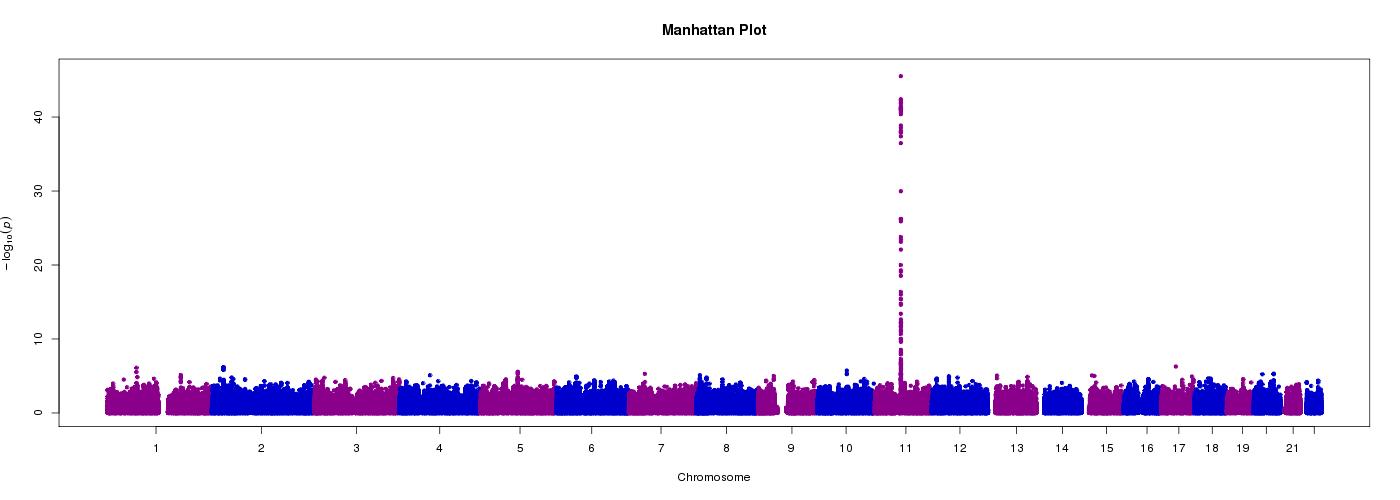


*FADS1/2*

Supplemental Figure S2. Quantile-Quantile plots for Chinese- and European-specific genome-wide association analyses. (A) 18:1n-9 in Chinese; (B) 18:1n-7 in Europeans; (C) 20:1n-9 in Europeans.

(A) (B) (C)


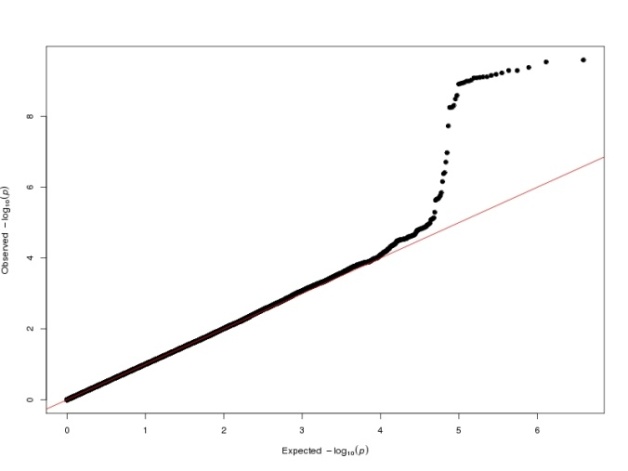

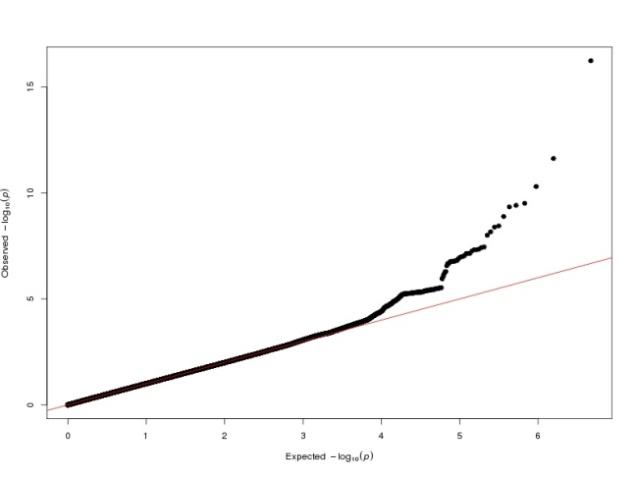

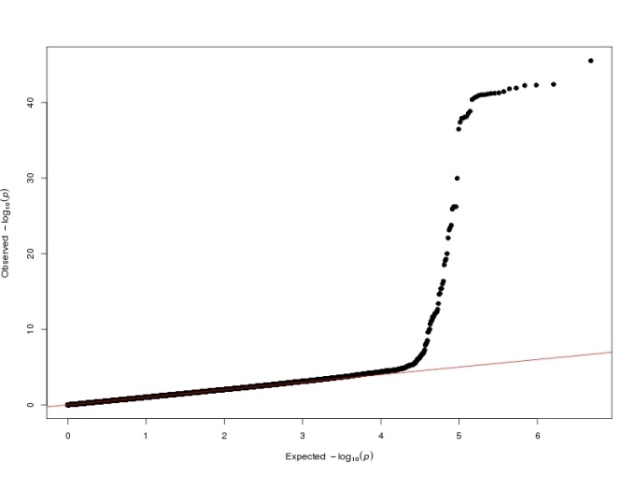


Supplemental Figure S3. Forest plots for meta-analysis of novel genome-wide associations. Within cohort effect sizes and 95%CIs were obtained from linear regression analysis using robust standard errors, and results were combined using inverse-variance weighted meta-analysis. (A) 18:1n-7, *PKD2L1*-rs603424, allele A; (B) 18:1n-7, *FADS1/2*-rs174528, allele C; (C) 20:1n-9, *FADS1/2*-rs174528, allele C; (D) 20:1n-9, *GCKR*-rs780094, allele T.

(A)


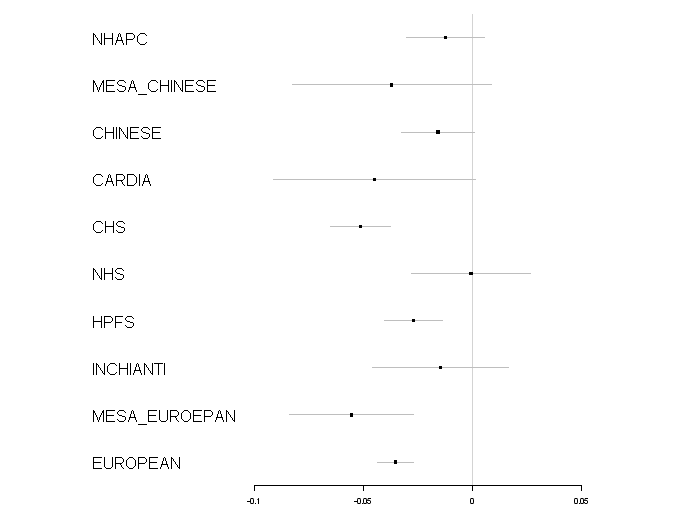


(B)


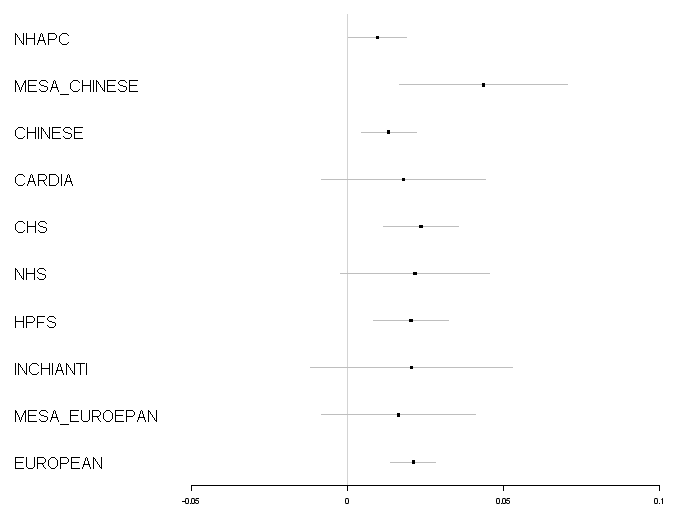


(C)


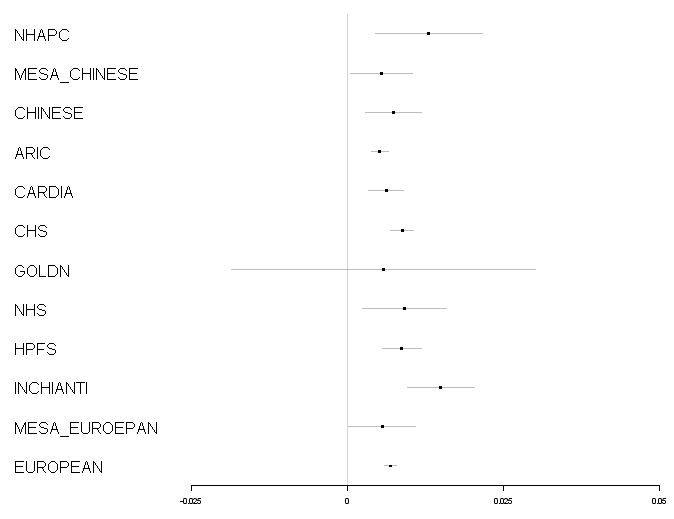


(D)


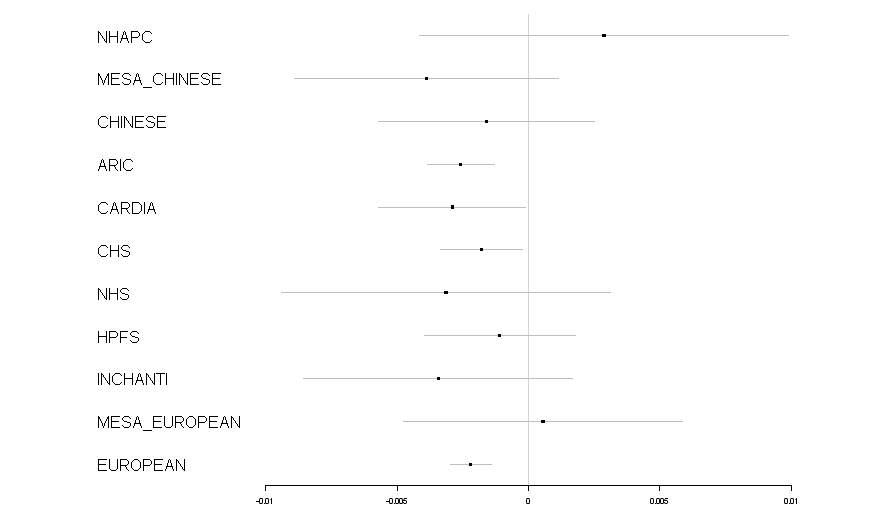

Supplement: Supplemental Data [file 10.1194_P071860_jlr.P071860-3.docx]
